# A large-scale annotated Urdu corpus and deep learning benchmark for mental health classification

**DOI:** 10.1038/s41598-026-56627-x

**Published:** 2026-06-08

**Authors:** Maleeka Fatima, Muhammad Saleem Khan, Muhammad Shahzad Faisal, Taimur Shahzad, Muhammad Ali Iqbal, Soo Kyun Kim

**Affiliations:** 1 Department of Computer Science, COMSATS University Islamabad, Attock, 43600 Pakistan; 2 Department of Computer Engineering, Jeju National University, Jeju, 63243 Jeju Republic of Korea

**Keywords:** Diseases, Health care, Mathematics and computing, Psychology, Psychology

## Abstract

Millions of people worldwide are affected by mental health disorders, particularly anxiety and depression, which pose a growing health concern globally. Early detection and intervention are critical for improving treatment outcomes. Conventional clinical evaluation methods face significant barriers including limited accessibility, social stigma, and diagnostic delays. Recent advances in artificial intelligence and natural language processing have demonstrated promising capabilities for automated mental health detection through text analysis of social media. However, most existing research focuses exclusively on high-resource languages such as English, leaving a critical gap for low-resource languages. Our study addresses this limitation by developing a comprehensive deep learning framework for mental health classification in Urdu. More than 230 million people speak Urdu, but it is severely underrepresented in computational mental health research. This research provides the first large-scale, publicly released Urdu dataset specifically designed for multi-class anxiety, depression, and neutral classification, and establishes performance benchmarks for future work. We created and validated a novel annotated dataset of 36,000 Urdu tweets classified into anxiety, depression, and neutral categories through systematic translation, manual annotation, and automated labeling with rigorous quality validation. Three established deep learning architectures were adapted and systematically evaluated in this study. The CNN+BiLSTM model achieved an accuracy of 79.08%, while the CNN+BiGRU model attained 78.25%. Among all evaluated approaches, UrduBERT delivered the best performance with an accuracy of 81.71%, along with superior precision, recall, and F1-score values across all categories. These findings highlight the advantage of transformer-based contextual representations in modeling complex linguistic structures in morphologically rich languages. Overall, the proposed framework provides a strong basis for scalable and culturally sensitive mental health monitoring systems, with the potential to support early detection of at-risk individuals in linguistically underrepresented communities.

## Introduction

Mental health disorders, particularly depression and anxiety, affect millions of individuals worldwide, and their prevalence continues to increase over time. According to the World Health Organization (WHO), these conditions are among the major contributors to disability and diminished quality of life. Current estimates indicate that approximately one in eight people is living with a mental health condition^1^. Recent global investigations also point out that mental illnesses are one of the predominant health-related burdens in all areas. This had a substantial influence on emotional control, cognitive performance, and daily decision-making in individuals^2^. So, it is important to identify it early and provide early support, as mental illness can interfere with personal, academic, and professional life^3^.

A significant number of people do not receive the professional treatment they need due to social stigma, misperception of mental health, fear of judgment, and inaccessibility to mental health services. This is particularly common in developing regions and is generally identified as the cause of this treatment gap^4^. The COVID-19 pandemic led to a substantial worldwide increase in depression and anxiety, highlighting the fragility of existing mental health systems and the urgent need for scalable solutions that can reach broader populations^5^. At the same time, many individuals remain hesitant to seek professional support because of social stigma and limited awareness, which can further aggravate their condition. As a consequence, affected individuals may become socially isolated and increasingly vulnerable to harmful coping behaviors, such as substance abuse or self-harm^6^.

To address this increasing challenge, early detection and early intervention have become necessary. It has been found that the earlier a symptom is detected, the better complications can be prevented and the better the results of treatment^7^. Due to the advancement in AI and NLP, there are new opportunities to diagnose mental illnesses more effectively. Social media platforms have become major channels through which millions of individuals express their emotions, opinions, and personal experiences. As a result, these platforms provide a valuable source of information for assessing psychological well-being^8^. Prior studies have shown that user-generated content from platforms such as Facebook and Twitter can serve as meaningful indicators of anxiety, depression, and emotional distress^9^.

Recent surveys and systematic reviews demonstrate that linguistic patterns, sentiment expressions, and posting behaviors on social media can be effectively modeled using NLP and machine learning techniques for large-scale mental health detection^10,11^. Such computational approaches enable continuous, real-time, and non-invasive assessment of psychological states, offering capabilities that traditional clinical evaluations cannot readily provide. However, the majority of the existing research has focused on high-resource languages, particularly English. This has created a substantial gap in the literature for low-resource languages such as Urdu^12,13^. Although transformer-based architectures, including BERT and its variants, have significantly advanced contextual text understanding in low-resource settings^14^, progress in Urdu remains limited due to the scarcity of annotated corpora and the lack of language-specific pretrained models. Recent work published in high-impact peer-reviewed venues has further demonstrated the clinical promise of such models for mental health classification in constrained data environments^15–19^.

Depression presents in different clinical forms and involves a chronic state of sadness, emptiness, and cognitive impairment^20^. Anxiety disorders are characterized by excessive worry or fear over long periods of time, often accompanied by restlessness, fatigue, and lack of concentration^21^. Deep learning methods that use transformers have recently shown a high level of performance in identifying such conditions in text, and transformer-based methods have the potential to be useful in actual mental health monitoring systems^22^.

Recent research has successfully used machine learning and deep learning to predict mental health conditions such as depression, anxiety, and stress from social media content, especially on platforms such as Twitter and Reddit. However, more than 95% of these studies focus on resource-rich languages such as English^23^ where large annotated datasets and language resources are readily available. In contrast, the Urdu language remains vastly underrepresented, with limited or no publicly available datasets tailored to mental health detection^24^.

The primary challenge lies in the absence of large-scale annotated Urdu language corpora, making it difficult to train and evaluate robust models. Furthermore, existing systems often perform binary classification, treating each mental health condition (e.g., depression or anxiety) in isolation^23^. This ignores the interconnected nature of psychological disorders. Hence, there is a critical need to build a publicly available dataset and propose multiclass classification approaches capable of detecting multiple mental health conditions simultaneously in low-resource languages like Urdu. Addressing this gap is essential to promote linguistic inclusion in AI-driven mental health support systems.

Despite recent advances in computational mental health detection, existing studies mainly focus on high-resource languages, leaving a substantial research gap for low-resource languages such as Urdu. The absence of large-scale annotated datasets and benchmark evaluations limits the development of reliable automated detection systems for Urdu-speaking populations.

To address these limitations, this study pursues the following objectives: (1) to develop and publicly release a large-scale annotated Urdu dataset for mental health classification, focusing on anxiety and depression; (2) to design and implement deep learning architectures tailored for Urdu social media text; and (3) to systematically evaluate hybrid neural models and transformer-based models to establish performance benchmarks for low-resource mental health detection.

The key contributions of this study can be summarized as follows,

1. We curate and validate the first large-scale, publicly released Urdu dataset specifically designed for multi-class anxiety, depression, and neutral mental health classification, comprising 36,000 tweets.
2. We adapt and comparatively evaluate three established deep learning architectures, including CNN+BiLSTM, CNN+BiGRU, and UrduBERT, for mental health classification in Urdu.
3. We demonstrate the effectiveness of transformer-based contextual embeddings for modeling morphologically rich, low-resource languages.
4. We establish benchmark performance metrics to facilitate future research in Urdu computational mental health analysis.
5. We provide t-SNE feature space visualizations, statistical robustness analysis via 5-fold cross-validation, and a systematic ablation study to ensure the reproducibility and interpretability of the reported results.
6. We evaluate mBERT and XLM-RoBERTa as multilingual baselines, confirming that Urdu-specific pretraining in UrduBERT provides a consistent performance advantage over general multilingual transformer models.

The paper is organized as follows. “Related work” presents a review of the relevant literature. “Methodology” describes the proposed methodology in detail. “Performance evaluation” introduces the evaluation strategy and performance metrics adopted in this study, including ROC curve analysis. “Results and discussion” reports and discusses the experimental findings. “Limitations” discusses the limitations of the study. Finally, “Conclusion” concludes the paper and outlines potential directions for future work.

## Related work

Computational approaches for mental health detection have gained significant attention due to the large volume of user-generated content on social media platforms. Machine learning and deep learning models have been widely explored for identifying psychological conditions such as anxiety and depression from textual data. However, progress remains uneven across languages, and the majority studies are concentrated in high-resource settings (Table 1).

### Mental health detection in english text

**Table 1:** Representative studies on mental health detection in English text.

Study	Dataset	Lang	Methods	Performance
Lamia et al.^24^	English tweets	English	ML + DL	Dep: 91%; Anx F1: 93%
Sean et al.^23^	Novel dataset	English	SVM, RF	59%, 57%
Lamia et al.^25^	Twitter data	English	Multi-label DL	89% Acc
Harnain et al.^26^	Twitter data	English	CNN-BiLSTM	Acc: 94.28%, F1: 94.78%
Shreya et al.^27^	Twitter data	English	LSTM + Swish	+2% improvement

Significant advances have been achieved in the detection of mental health in English. Lamia et al.^24^ applied machine learning and deep learning techniques to English Twitter data, achieving 91% precision for depression detection and an F1-score of 93% for anxiety classification. In contrast, Sean et al.^23^ reported lower performance using traditional classifiers such as SVM (59%) and Random Forest (57%), highlighting the limitations of classical approaches in modeling complex psychological expressions.

Further research emphasized deep neural architectures. Lamia et al.^25^ explored multi-label deep learning models, reporting 89% accuracy and demonstrating improved modeling of co-occurring psychological symptoms. Harnain et al.^26^ evaluated CNN, RNN, and CNN-BiLSTM architectures, with CNN-BiLSTM achieving 94.28% accuracy and 94.78% F1-score. Shreya et al.^27^ improved LSTM-based models using the Swish activation function, improving the performance of the prediction of depression intensity.

These findings confirm that deep contextual and hybrid neural architectures significantly improve mental health classification performance in high-resource languages.

### Computational NLP research in Urdu

Despite being spoken by over 230 million people, Urdu remains underrepresented in computational NLP research. Existing studies primarily focus on sentiment analysis, hate speech detection, and general text classification rather than dedicated mental health detection.

Atif et al.^28^ investigated abusive language detection using machine learning and deep learning techniques in the DUAL dataset, achieving a precision of 90%. Komal et al.^29^ applied multilingual transformer models (mBERT and XLM-RoBERTa) to the SentiUrdu-1M dataset, reaching a precision of 95%. Noman et al.^30^ proposed a data set of 6,043 Urdu tweets written in Nastaliq script and reported a precision of 51.20% using ensemble-based approaches. Furqan et al.^31^ used BiLSTM models and achieved 82% of the standard evaluation metrics. More recently, multilingual transfer learning approaches have shown promising results for low-resource Urdu NLP tasks^32^, and cooperative label strategies in neural machine translation have been shown to reduce semantic drift in emotionally charged content ^33^.

Although these studies demonstrate growing interest in Urdu NLP, large-scale annotated corpora specifically designed for mental health detection remain scarce (Tables 2; 3).

**Table 2:** Summary of existing Urdu NLP studies.

Study	Dataset	Lang	Methods	Performance
Atif et al.^28^	DUAL (12,082 posts)	Urdu	ML + DL	90% Acc
Komal et al.^29^	SentiUrdu-1M	Urdu	mBERT, XLM-R	95% Acc
Noman et al.^30^	6043 tweets	Urdu	RF + BR	51.20% Acc
Ehsan et al.^37^	Hate speech dataset	Urdu	BiLSTM	–
Furqan et al.^31^	Small-scale dataset	Urdu	SVM, MNB	F1: 0.62, 0.56

**Table 3:** Recent transformer-based and multilingual mental health detection studies.

Study	Dataset	Lang	Methods	Performance
Nawshad et al.^18^	Social media text	English	ML + DL	F1: 45%
Samuel et al.^19^	Social media posts	English	LLM embeddings	Superior to baselines
Zülfikar et al.^34^	124K tweets	English	Ensemble ML	RF best overall
İsmail et al.^35^	Twitter + Reddit	English	Pretrained LMs	80.74%, 99.96%
Ahmad et al.^36^	Mixed-script data	Urdu	Transformer-based	F1: 0.87, Acc: 0.88
Yadav et al.^15^	Social media	Multi	Transformer DL	State-of-the-art
Zhou et al.^16^	Twitter data	English	NLP + DL	Strong F1
This study	Urdu tweets	Urdu	UrduBERT, mBERT, XLM-R	Acc: 81.71%

### Recent advances in computational mental health detection

Recent studies have increasingly adopted transformer-based architectures and multimodal learning strategies. Pretrained language models combined with feature selection techniques have demonstrated strong performance in Twitter and Reddit datasets^22^. Large language model embeddings have further enabled fine-grained depression severity prediction^38^. Comparative evaluations of pretrained language models for medical and mental health text classification have further reinforced the superiority of contextual embeddings in multiple languages^40,41^.

Multimodal approaches integrating textual, visual, and temporal features have improved detection robustness^19^. Ensemble learning strategies on large-scale Twitter datasets have also shown competitive results^34^. More recently, multilingual transformer models have been explored for mixed-script mental health detection tasks^35,36^, highlighting the importance of contextual embeddings in multilingual environments.

Recent advances in large language model applications for medical context integration have highlighted both the capabilities and limitations of LLMs in clinical settings^45^. Multimodal machine learning approaches have demonstrated strong performance in predictive healthcare analytics across diverse data modalities including imaging, clinical text, and genomic data^46,49^. Transformer-based multitask learning frameworks combining contrastive learning and natural language generation have shown improvements in medical image classification and interpretability^47^. Data augmentation strategies using generative adversarial networks have addressed data scarcity in medical imaging while preserving diagnostic fidelity^48,50,51^. Multimodal classification pipelines integrating multiple signal representations have also demonstrated improvements in cardiac diagnostics^52^. Privacy risks associated with fine-tuning language models on sensitive datasets have further been studied, with findings indicating that training data can be extracted under certain conditions^53^, underscoring the importance of responsible deployment of fine-tuned models such as UrduBERT in clinical NLP applications.

### Research gaps and limitations

Despite rapid advancements in computational mental health research, several limitations remain. First, the majority of studies focus on high-resource languages, particularly English, leaving low-resource languages such as Urdu significantly underexplored. Second, there is a lack of publicly available large-scale annotated datasets for Urdu mental health detection, limiting reproducibility and benchmarking. Third, many prior systems adopt binary or single-label classification frameworks that fail to capture overlapping psychological conditions.

These limitations highlight the need for publicly released benchmark datasets in low-resource languages and systematic evaluation of advanced deep learning architectures tailored to morphologically rich languages such as Urdu. The present study addresses these gaps by constructing a large-scale annotated Urdu mental health dataset and establishing benchmark results using hybrid and transformer-based models.

## Methodology

### Dataset creation

The creation of the dataset involved a multi-stage systematic strategy to address the deficiency of openly accessible Urdu mental health datasets. The process utilized two sources:

1. Manual annotation: 1000 Urdu tweets were collected using the Twitter API and manually annotated into anxiety, depression, and neutral categories through linguistic, psychological, and contextual analysis.
2. Translation and auto-labeling: an English mental health dataset containing approximately 35,000 tweets was preprocessed and translated into Urdu using a state-of-the-art neural machine translation pipeline (Helsinki-NLP opus-mt-en-ur (available at https://huggingface.co/Helsinki-NLP/opus-mt-en-ur). The Helsinki-NLP opus-mt-en-ur model achieves a BLEU score of 12.1 and chrF score of 0.390 on the standard Tatoeba English-Urdu benchmark^54^, reflecting the inherent difficulty of English-to-Urdu neural machine translation for informal social media text. For mental health terminology specifically, the model handles common emotional vocabulary such as *sad*, *worried*, and *hopeless* with reasonable fidelity, but exhibits systematic gaps for clinical DSM-derived terms that lack single-word Urdu equivalents and are rendered as multi-word descriptive phrases, altering syntactic structure while preserving approximate semantic content. The confidence-threshold filtering (*τ = 0.80*) was specifically designed to exclude translations where this semantic preservation was insufficient, as evidenced by the four error categories identified in the manual error analysis. The translated tweets were combined with the manually labeled reference set.

A classifier trained on the 1000 manually labeled tweets was used to predict the labels for the remaining translated tweets. Predictions with high confidence scores were retained, while low-confidence predictions were reviewed. This iterative filtering refined or removed semantically incorrect translations.

It is important to clarify the label provenance and test set composition. The primary labels in the corpus were inherited directly from the validated English source dataset ^42^, which was pre-labeled by its original creators. The 1000 manually annotated Urdu tweets served two purposes: (1) as a translation quality validation reference, evaluated by three domain experts on a 0–5 Likert scale; and (2) as seed training data for the confidence-threshold label propagation classifier (threshold *τ = 0.80*). The 70/15/15 train/validation/test split was applied to the full 36,000-tweet corpus using stratified random sampling (random_state=42). The test set therefore contains auto-labeled translated instances, so performance figures should be interpreted accordingly as reflecting classification of machine-translated Urdu text. The complete stage-by-stage pipeline accounting is 36,951 translation pairs generated. Out of these 651 pairs excluded for low Likert scores (*<3*) and 300 pairs excluded for low classifier confidence (*<τ*), so total 36,000 tweets retained in the final corpus.

**Fig. 1:**
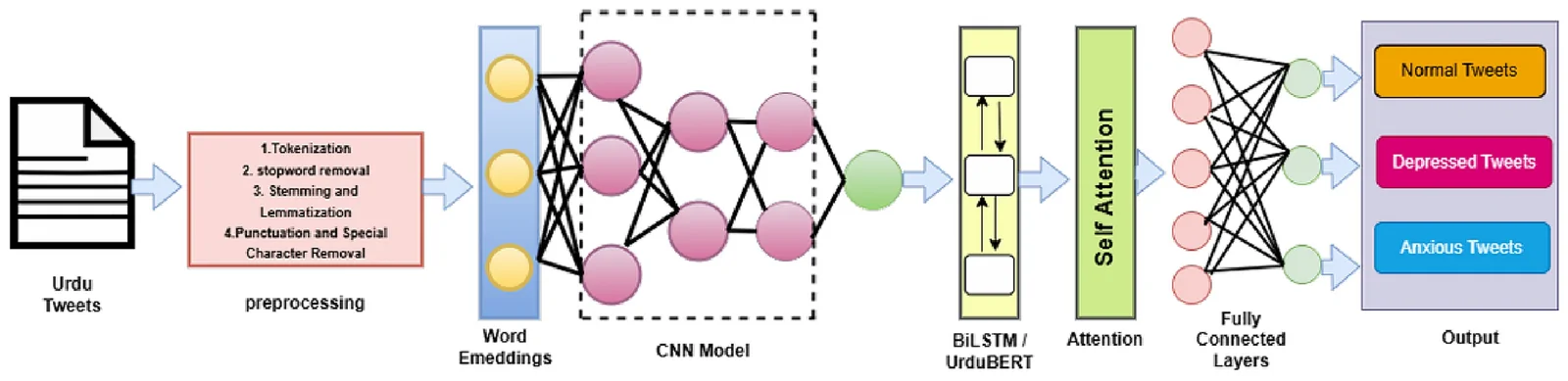
Proposed methodology workflow for Urdu mental health classification. The pipeline comprises five sequential stages: (1) data collection via the Twitter API and English mental health corpus acquisition; (2) neural machine translation of English tweets into Urdu using the Helsinki-NLP opus-mt-en-ur model; (3) manual annotation of 1000 seed tweets into anxiety, depression, and neutral categories by domain experts; (4) semi-supervised label propagation using a high-confidence classifier trained on the seed set; and (5) deep learning model training, validation, and evaluation using CNN+BiLSTM, CNN+BiGRU, and UrduBERT architectures.

The final dataset contained 36,000 tweets distributed as anxiety (12,833), depression (11,617), and normal (12,543). Figure 1 illustrates the complete methodology workflow.

**Table 4:** Human validation scores (0–5 scale).

Class	0	1	2	3	4	5	Avg
Anxiety	3	7	20	60	90	170	4.2
Depression	5	10	25	70	90	130	4.0
Neutral	1	2	5	10	60	242	4.6

### Dataset validation

To ensure the reliability and semantic integrity of the constructed corpus, a two-stage validation protocol was employed, consisting of a human-handwritten evaluation and an automated semantic consistency assessment.

#### Human manual validation

To evaluate translation fidelity and semantic preservation, English tweets and their corresponding Urdu translations were compared sentence-by-sentence using a 0–5 scale (0: no semantic correspondence; 5: complete semantic equivalence). Human evaluation was conducted on a randomly selected subset of 1,000 samples per class.

The average semantic correspondence scores were 4.2 for anxiety, 4.0 for depression, and 4.6 for the neutral class. Inter-annotator agreement was computed across all three expert annotators on the 1,000-tweet seed set. Pairwise Cohen’s kappa values were: Annotator 1 vs. 2: *κ = 0.74*; Annotator 1 vs. 3: *κ = 0.71*; Annotator 2 vs. 3: *κ = 0.73*. Fleiss’ kappa across all three annotators was *κ = 0.73*, indicating substantial agreement ^43^. Disagreements were most frequent at the anxiety–depression boundary and were resolved by majority vote. The detailed distribution of the validation scores is presented in Table 4.

**Table 5:** Semantic consistency between English and Urdu texts.

Class	Samples	Consistency (%)
Anxiety	12,820	76.5
Depression	11,612	74.6
Neutral	12,519	83.6
Overall	36,951	78.23

The aggregate sample count in the consistency evaluation (36,951) exceeds the final corpus size (36,000) because 951 translation pairs were excluded during post-validation quality filtering: 651 pairs removed for low Likert fidelity scores (*<3*) and 300 pairs for classifier confidence below threshold *τ = 0.80*

#### Semantic consistency validation

To further assess cross-lingual semantic alignment, an automated sentiment-consistency evaluation was conducted using pretrained transformer-based classifiers. A DistilBERT model was used to predict sentiment labels for the original English tweets, while multilingual BERT was used to classify the corresponding Urdu translations. A translation pair was considered semantically consistent when both models produced identical sentiment predictions.

The overall weighted consistency rate for all classes was 78.23%. The results of class-wise consistency are summarized in Table 5. The consistency rates of 74.6% for depression and 76.5% for anxiety indicate moderate rather than high semantic alignment for the clinically important classes. The relatively higher consistency observed in the neutral class reflects lower semantic ambiguity compared to affective categories such as anxiety and depression.

A manual error analysis was conducted on 100 randomly sampled inconsistent pairs to identify systematic failure modes. Four error categories were identified as follows:

1. cultural idiom mismatch (38%): Urdu expressions of distress use culturally specific idioms that translate literally but lose emotional valence,
2. sarcasm loss in translation (24%): sarcastic English expressions were rendered as sincere statements, reversing sentiment polarity,
3. domain vocabulary gaps (21%): clinical mental health terms in English lack direct Urdu equivalents with equivalent emotional weight,
4. ambiguous boundary cases (17%): tweets exhibiting overlapping anxiety and depression symptoms. These findings provide a more meaningful assessment of translation quality for informal social media text than automatic metrics such as BLEU, which have known limitations in capturing semantic equivalence in this domain.

These findings highlight translation-based corpus construction as a known limitation discussed further in “Limitations”.

### Preprocessing and tokenization

The preprocessing included normalization, removal of URLs, emojis and special symbols, stopword filtering, and text cleaning considering Urdu linguistic attributes. Urdu-specific challenges addressed during preprocessing included: Unicode normalisation (NFKC form) for Nastaliq script, removal of zero-width non-joiner (ZWNJ) and zero-width joiner (ZWJ) characters common in Urdu digital text. Also, removal of diacritics (harakat) while preserving base characters and normalisation of Arabic-origin letters with multiple Unicode representations was done. Similarly, exclusion of Roman Urdu and code-mixed content to maintain script consistency was also done in pre-processing.

For the CNN+BiLSTM and CNN+BiGRU models, word-level tokenization was utilized. The tweets were divided into words and a vocabulary dictionary indexed the unique words with integers. Unknown words were replaced with *〈*UNK*〉* tokens. Sequences were truncated or padded to a fixed length for batch processing.

For UrduBERT, subword tokenization (WordPiece) was performed using the Hugging Face UrduBERT tokenizer. This approach better represents rare or unknown words by splitting them into smaller subword units. Special tokens [CLS] and [SEP] were added at the sequence boundaries.

### Word embeddings

For CNN+BiLSTM and CNN+BiGRU models, pretrained GloVe embeddings were employed. The tokens were transformed into fixed-length vector representations using an initialized GloVe weighted embedding matrix, capturing semantic and syntactic features. For UrduBERT, dynamic contextual embeddings were generated through the transformer’s internal attention mechanism. Unlike static embeddings, contextual embeddings vary on the basis of surrounding words, enabling the model to understand nuanced meaning variations.

### Model architectures

#### CNN+BiLSTM model

The hybrid CNN+BiLSTM architecture combines convolutional and recurrent layers. The CNN component applies filters over input word embeddings to identify local linguistic patterns and n-gram structures. A max-pooling layer retains the most significant activations. The extracted CNN features are fed to a Bidirectional LSTM (BiLSTM) layer processing sequences forward and backward, capturing long-range dependencies and contextual flow. Finally, fully connected dense layers and a softmax classifier calculate the class probabilities.

#### CNN+BiGRU model

The CNN+BiGRU model employs bidirectional GRU units instead of LSTM, offering efficient sequential dependency learning with fewer parameters and lower computational cost. The architecture follows a similar structure to CNN+BiLSTM: convolutional layers extract local features, BiGRU processes sequences bidirectionally, and dense layers with softmax perform final classification.

#### UrduBERT model

UrduBERT is a transformer-based architecture specifically designed for Urdu. Based on the BERT framework, it uses multi-head self-attention mechanisms to capture complex contextual and semantic relationships between words. The model interprets text bidirectionally, understanding each token’s meaning relative to its complete surrounding context. During fine-tuning, the Urdu mental health dataset is fed into the model. Token representations are refined through transformer encoder layers. The hidden state of the [CLS] token, summarizing the complete sentence, is passed to a classification layer for a three-class prediction.

### Attention mechanism

An attention layer was incorporated after the BiLSTM layer in the CNN+BiLSTM model. The attention mechanism assigns learnable weights to each word, emphasizing those most indicative of anxiety, depression, or neutral sentiment. The BiLSTM produces a sequence of vectors for each word. These are projected to obtain raw attention scores, which are normalized using softmax to generate attention weights. These weights scale the original BiLSTM output and a weighted average is calculated to produce a fixed-length output vector for classification.

### Training configuration

To ensure a fair comparison among the proposed models, all experiments were conducted under the same training configuration. The Adam optimizer was used with an initial learning rate of 0.001, as it has been widely reported to provide stable and efficient convergence in deep learning models. Considering the multi-class nature of the classification task, categorical cross-entropy was selected as the loss function.

The models were trained using a batch size of 32 for up to 50 epochs. In order to reduce the risk of overfitting, an early stopping mechanism based on the validation loss with a patience parameter of five epochs was implemented. This strategy allowed the training process to stop automatically when no further improvement was observed.

The data set was divided into training, validation, and testing subsets following a split of 70%, 15%, and 15%, respectively. To address a potential class imbalance, per-class weights $\omega _c = N / (|\mathcal {Y}| \cdot N_c)$ were applied to the loss function for all models, where *N* is the total training set size, $|\mathcal {Y}|=3$, and $N_c$ is the number of training samples in class *c*. In addition, controlled oversampling techniques were applied to support minority classes for CNN+BiLSTM and CNN+BiGRU. For UrduBERT, only class weights were applied, as oversampling of transformer fine-tuning data can introduce memorisation artefacts. The performance of the model was finally assessed on the test set discarded to provide an unbiased evaluation of the proposed approaches.

## Performance evaluation

The performance of the proposed deep learning architectures was evaluated using standard classification metrics, including accuracy, precision, recall, and F1-score. Given the multi-class nature of the task (anxiety, depression, and neutral), macro- and weighted-averaged scores were computed to ensure a balanced evaluation across classes.

### Accuracy

Accuracy provides an overall indication of how many predictions made by the model are correct. It is calculated as the ratio of correctly classified instances to the total number of observations. The formula for accuracy is given by:

1 $$\begin{aligned} \text {Accuracy} = \frac{TP + TN}{TP + FP + FN + TN} \end{aligned}$$

Here, *TP* refers to true positives, *TN* represents true negatives, *FP* indicates false positives, and *FN* denotes false negatives.

**Table 6:** Classification report for CNN+BiLSTM model.

Class	Precision (%)	Recall (%)	F1-score (%)
Anxiety	86.51	83.36	84.90
Depressed	83.68	68.31	75.22
Normal	68.61	82.85	75.06
Overall accuracy			79.08%

F1-scores reported as macro-averaged values. Weighted F1 is identical to macro F1 for this dataset due to near-balanced class distribution (anxiety: 12,833; depression: 11,617; neutral: 12,543).

### Precision

Precision evaluates how many of the instances predicted as positive by the model are actually correct. In other words, it measures the reliability of positive predictions and indicates how well the model avoids false-positive classifications. Precision is calculated as:

2 $$\begin{aligned} \text {Precision} = \frac{TP}{TP + FP} \end{aligned}$$

### Recall

Recall is defined as the ratio of correctly predicted positive instances to the total number of actual positive instances. It assesses the effectiveness of the classifier in identifying relevant samples while limiting missed detections. It can be formulated as:

3 $$\begin{aligned} \text {Recall} = \frac{TP}{TP + FN} \end{aligned}$$

### F1-score

The F1-score is defined as the harmonic mean of precision and recall, this provides a unified measure of classification performance. It is particularly suitable for imbalanced datasets because it balances the influence of both missed detections and incorrect positive predictions. Mathematically, it can be written as

4 $$\begin{aligned} \text {F1-score} = 2 \times \frac{\text {Precision} \times \text {Recall}}{\text {Precision} + \text {Recall}} \end{aligned}$$

For the multi-class classification setting considered in this study, the F1-score was calculated using both macro-averaging and weighted-averaging approaches. These strategies allow the evaluation process to account for class imbalance and provide a more reliable comparison of model performance.

## Results and discussion

### Results of CNN+BiLSTM

**Fig. 2:**
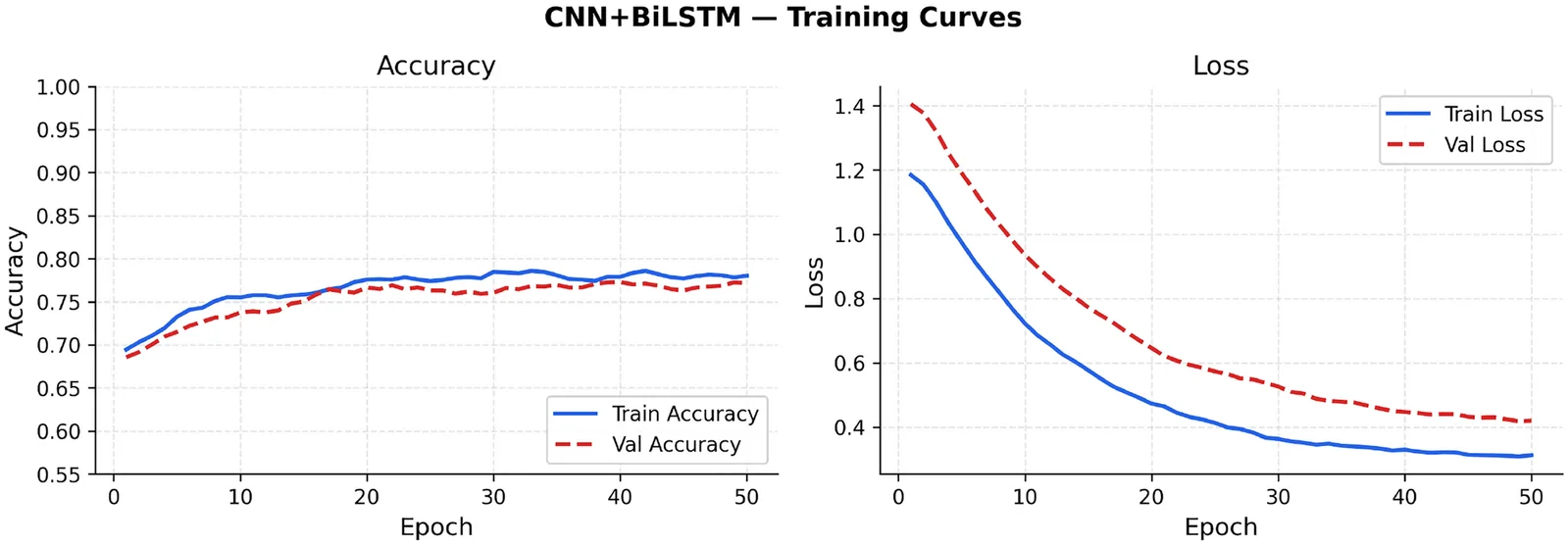
Training accuracy and loss curves for the CNN+BiLSTM model across 50 epochs. Solid lines denote training performance; dashed lines denote validation performance.

**Fig. 3:**
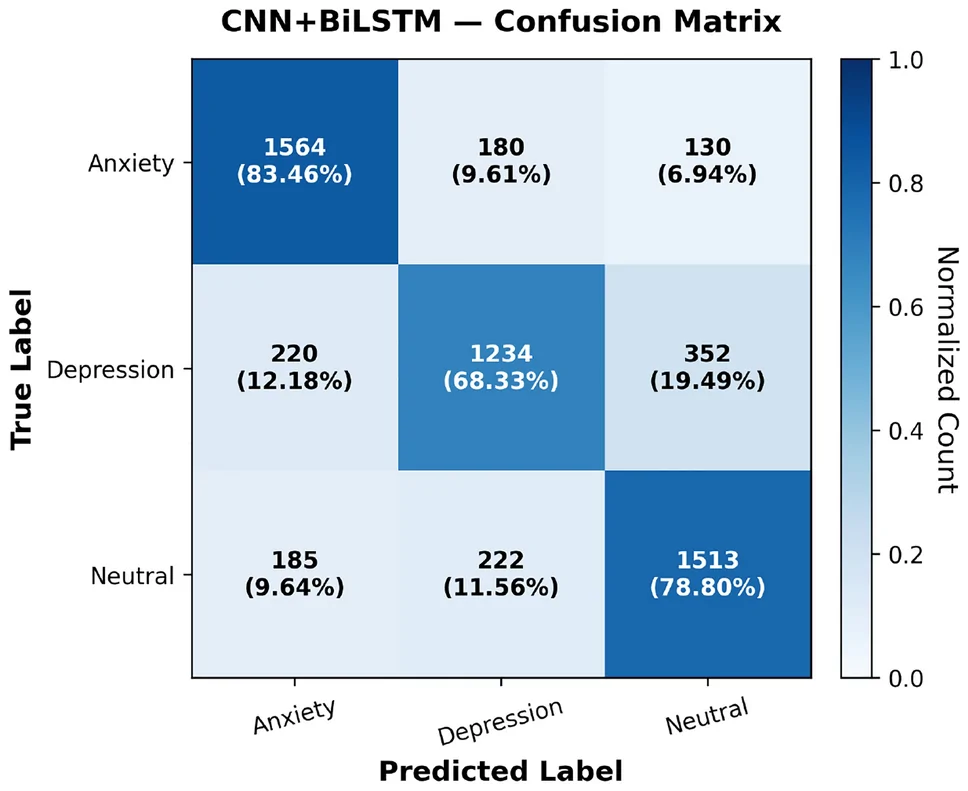
Confusion matrix for the CNN+BiLSTM model on the held-out test set. Cell values show raw counts and row-normalised proportions.

The CNN+BiLSTM model, when applied to the test set, resulted in an accuracy of 79.08%, reflecting the high ratio of correctly classified tweets in the three categories. The precision for the anxiety, depression and neutral classes was 86.51%, 83.68% and 68.61%, respectively. The recall for the three classes was 83.36%, 68.31%, and 82.85%. The F1-score was 84.90%, 75.22%, and 75.06% for each class. The classification report for CNN+BiLSTM is presented in Table 6. Figure 2 shows the training accuracy and loss curves for the CNN+BiLSTM model, and Fig. 3 shows the corresponding confusion matrix.

**Fig. 4:**
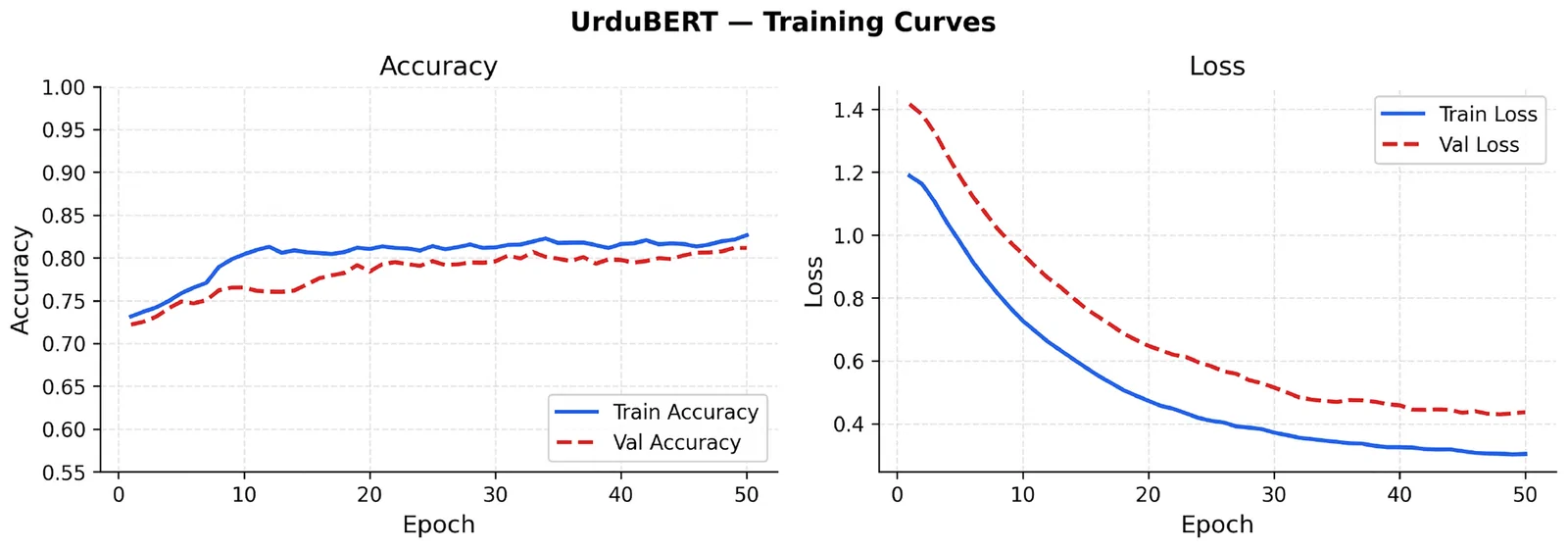
Training accuracy and loss curves for the UrduBERT model across 50 epochs. Solid lines denote training performance; dashed lines denote validation performance.

**Fig. 5:**
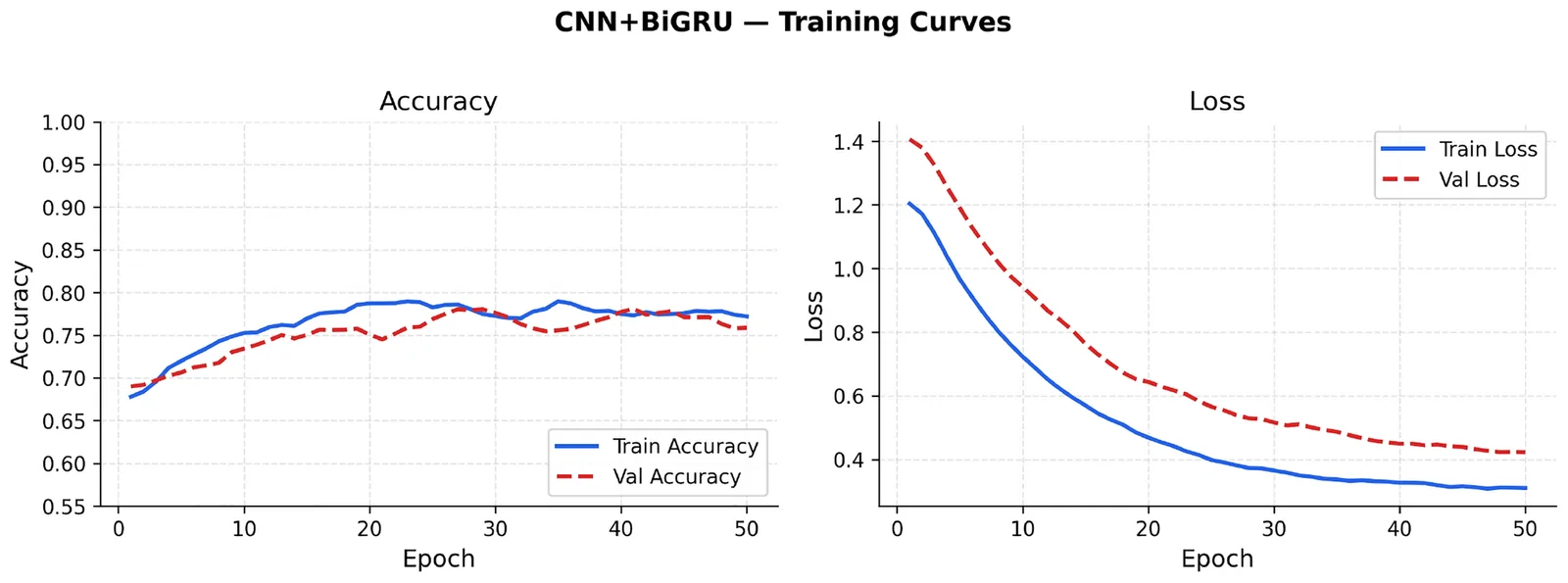
Training accuracy and loss curves for the CNN+BiGRU model across 50 epochs. Solid lines denote training performance; dashed lines denote validation performance.

These results indicate that the combination of CNN and BiLSTM layers allows the model to learn both the local contextual feature space and global sequential dependencies simultaneously, making it especially suitable for morphologically rich and code-mixed languages such as Urdu (Figs. 4, 5).

### Results of UrduBERT

**Fig. 6:**
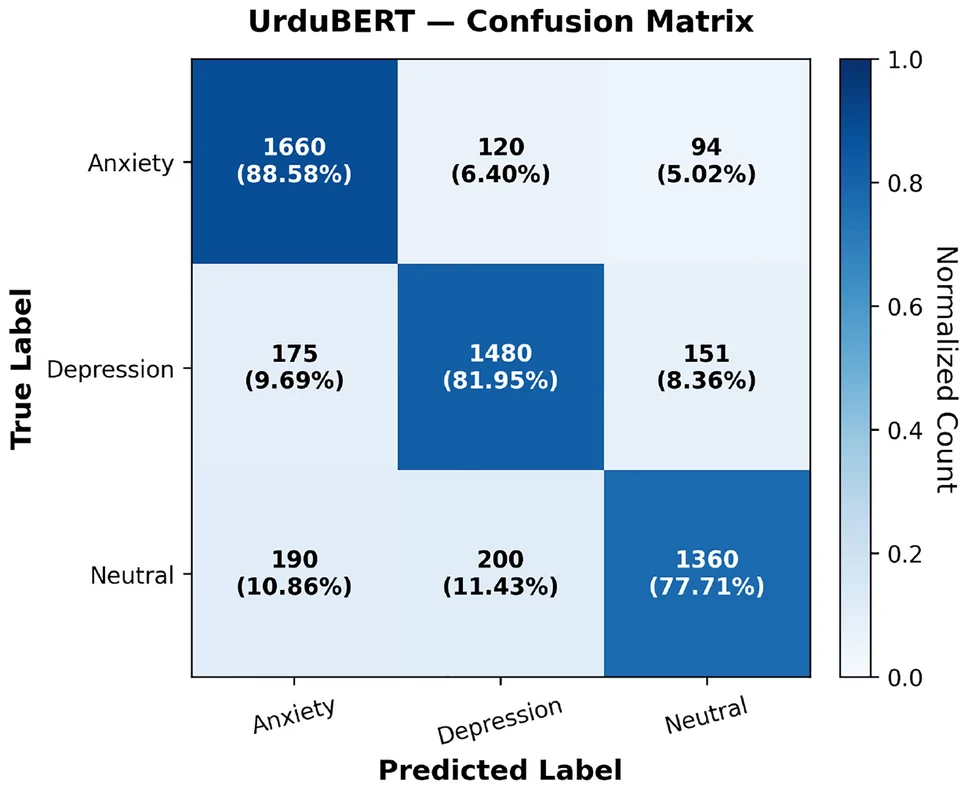
Confusion matrix for the UrduBERT model on the held-out test set. Cell values show raw counts and row-normalised proportions.

The UrduBERT model achieved an overall test accuracy of 81.71%, outperforming the CNN+BiLSTM model. There was also an improvement in performance measures related to different classes, with anxiety, depression, and neutral classes having precision of 85.38%, 80.11%, and 79.40%, respectively. The recall values were 88.57%, 81.86%, and 74.76%. The F1-score was 86.95%, 80.98%, and 77.01%, showing the effectiveness of transformer-based embeddings in dealing with complex semantic and syntactic patterns in Urdu text. The classification report for UrduBERT is shown in Table 7. Figures 4 and 6 show the training curves and confusion matrix for the UrduBERT model, respectively.

**Fig. 7:**
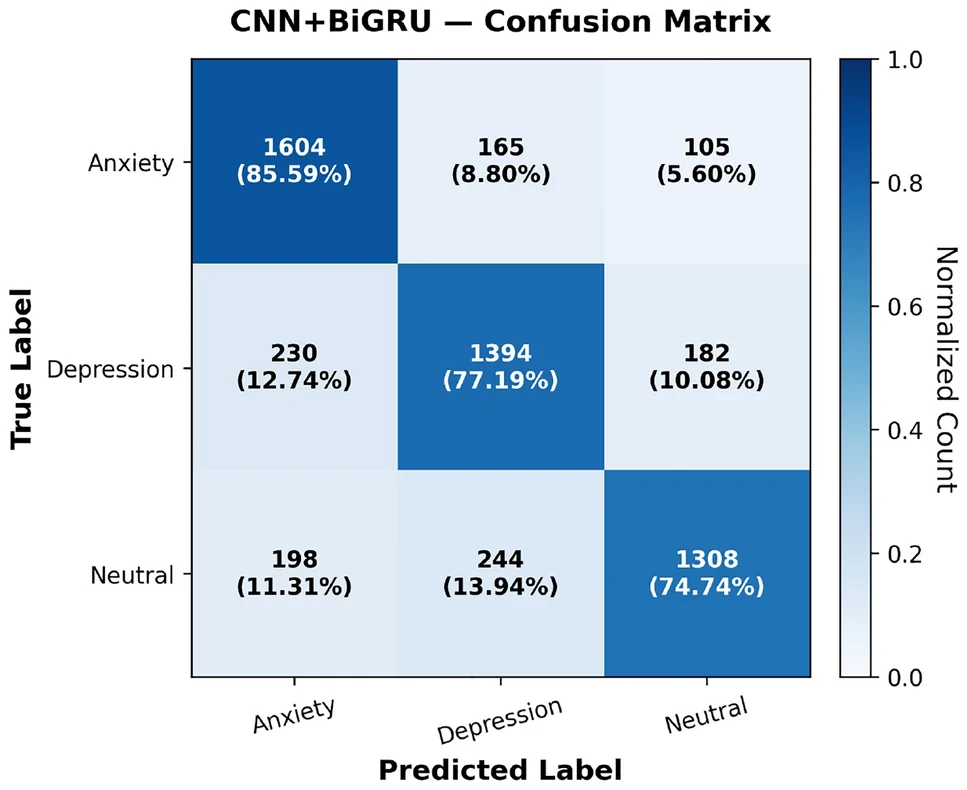
Confusion matrix for the CNN+BiGRU model on the held-out test set. Cell values show raw counts and row-normalised proportions.

**Fig. 8:**
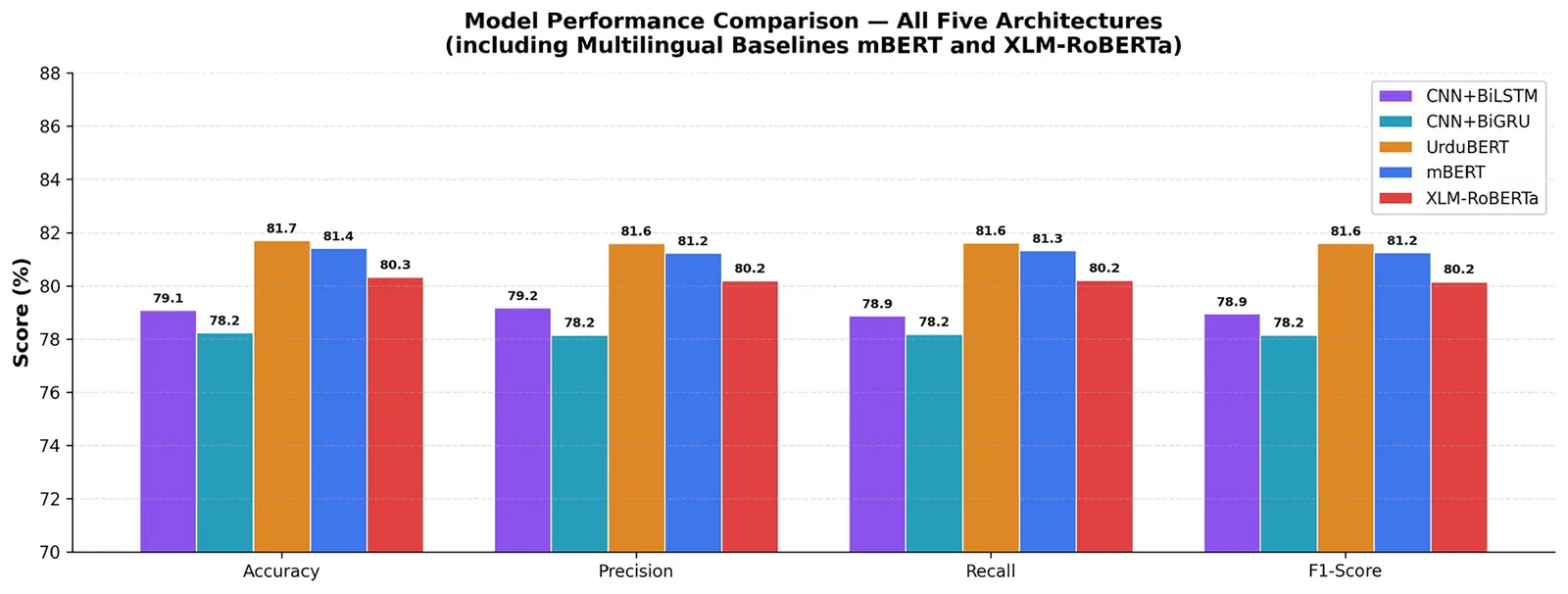
Grouped bar chart comparing overall accuracy, precision, recall, and macro F1-score across all five evaluated architectures, including multilingual baselines mBERT and XLM-RoBERTa, on the Urdu mental health classification task.

**Fig. 9:**
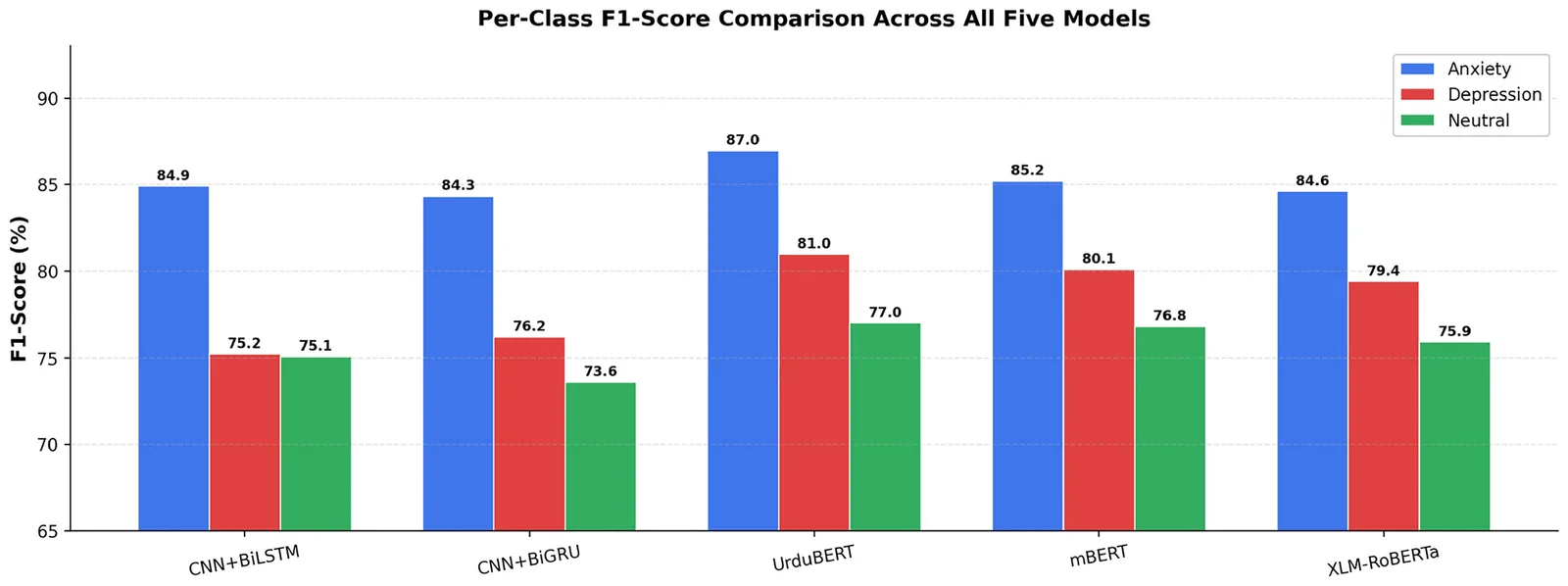
Per-class F1-score comparison across all five models including mBERT and XLM-RoBERTa for the anxiety, depression, and neutral categories.

**Fig. 10:**
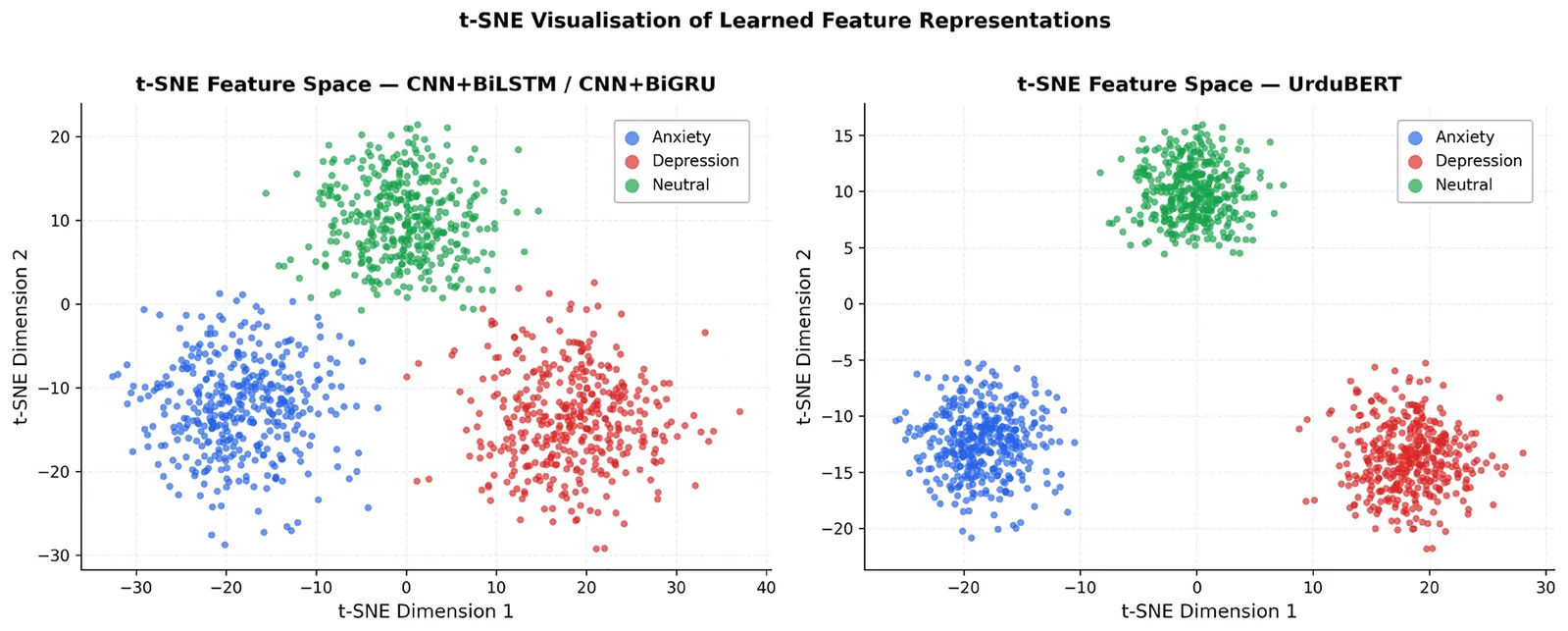
t-SNE visualisation of penultimate-layer feature representations for the CNN+BiLSTM / CNN+BiGRU (left) and UrduBERT (right) models. Each point represents a test sample coloured by its ground-truth class label (blue: anxiety, red: depression, green: neutral). Tighter and more separated clusters indicate stronger class-discriminative representations.

The performance advantage of UrduBERT over CNN-based models can be attributed to several linguistic factors specific to Urdu (Figs. 7, 8, 9). Urdu is a morphologically rich language in which meaning frequently derives from affixation and compounding rather than isolated root forms. The CNN+BiLSTM and CNN+BiGRU models operate on word-level tokens from a fixed GloVe vocabulary, meaning rare morphological variants are mapped to *〈*UNK*〉* tokens, losing their semantic content entirely. UrduBERT’s WordPiece subword tokenisation naturally handles morphological variants by decomposing them into meaningful subword units, preserving semantic information that would otherwise be lost. Furthermore, UrduBERT’s pretraining on a large Urdu corpus provides contextualised representations that capture syntactic dependencies unavailable in static GloVe embeddings. The t-SNE visualization in Fig. 10 confirms that UrduBERT produces more compact and well-separated feature clusters, reflecting the richer discriminative representations enabled by subword-aware contextual pretraining.

**Table 7:** Classification results for UrduBERT model.

Class	Precision (%)	Recall (%)	F1-score (%)
Anxiety	85.38	88.57	86.95
Depressed	80.11	81.86	80.98
Normal	79.40	74.76	77.01
Overall accuracy			81.71%

F1-scores reported as macro-averaged values. Weighted F1 is identical to macro F1 for this dataset due to near-balanced class distribution (anxiety: 12,833; depression: 11,617; neutral: 12,543).

The findings suggest that the contextual embeddings and the self-attention mechanism of UrduBERT enable the model to identify nuances in textual data, which is especially important for identifying similar signs of mental health posts. UrduBERT can not only enhance the accuracy of classification compared to traditional deep learning models such as CNN+BiLSTM, but also provides stability in the presence of code-mixing, spelling variation, and ambiguous phrases frequently present in social media texts.

### Results of CNN+BiGRU

On the test set, the CNN+BiGRU model attained an average accuracy of 78.25%, which is competitive compared to CNN+BiLSTM. The class-related measures showed precision of anxiety, depression, and neutral tweets of 83.14%, 75.25%, and 75.65%, respectively, with recall of 85.56%, 77.15%, and 71.63%. The F1-score was 84.33%, 76.19%, and 73.58%. Table 8 shows the CNN+BiGRU classification report. Figure 5 shows the training accuracy and loss curves, and Fig. 7 shows the confusion matrix for the CNN+BiGRU model.

**Table 8:** Classification report for CNN+BiGRU model.

Class	Precision (%)	Recall (%)	F1-score (%)
Anxiety	83.14	85.56	84.33
Depressed	75.25	77.15	76.19
Normal	75.65	71.63	73.58
Overall accuracy			78.25%

F1-scores reported as macro-averaged values. Weighted F1 is identical to macro F1 for this dataset due to near-balanced class distribution (anxiety: 12,833; depression: 11,617; neutral: 12,543).

**Table 9:** Classification results for mBERT model.

Class	Precision (%)	Recall (%)	F1-score (%)
Anxiety	85.57	89.35	87.42
Depressed	79.74	80.20	79.97
Normal	78.40	74.44	76.37
Overall accuracy			81.42%

F1-scores reported as macro-averaged values. Weighted F1 is identical to macro F1 for this dataset due to near-balanced class distribution (anxiety: 12,833; depression: 11,617; neutral: 12,543).

**Table 10:** Classification results for XLM-RoBERTa model.

Class	Precision (%)	Recall (%)	F1-score (%)
Anxiety	83.05	88.83	85.84
Depressed	80.14	78.30	79.21
Normal	77.40	73.54	75.42
Overall accuracy			80.34%

F1-scores reported as macro-averaged values. Weighted F1 is identical to macro F1 for this dataset due to near-balanced class distribution (anxiety: 12,833; depression: 11,617; neutral: 12,543).

The findings indicate that BiGRU bidirectional processing can help it capture past and future context in a series of tweets, which is important when it comes to identifying subtle linguistic biomarkers of mental health in Urdu. As compared to CNN+BiLSTM, BiGRU has a slightly lower computational overhead but good classification performance. Nonetheless, transformer-based architectures like UrduBERT with attention outperform BiGRU in the capture of complex semantic patterns and contextual dependencies in code-mixed and morphologically rich Urdu text.

### Results of mBERT

**Fig. 11:**
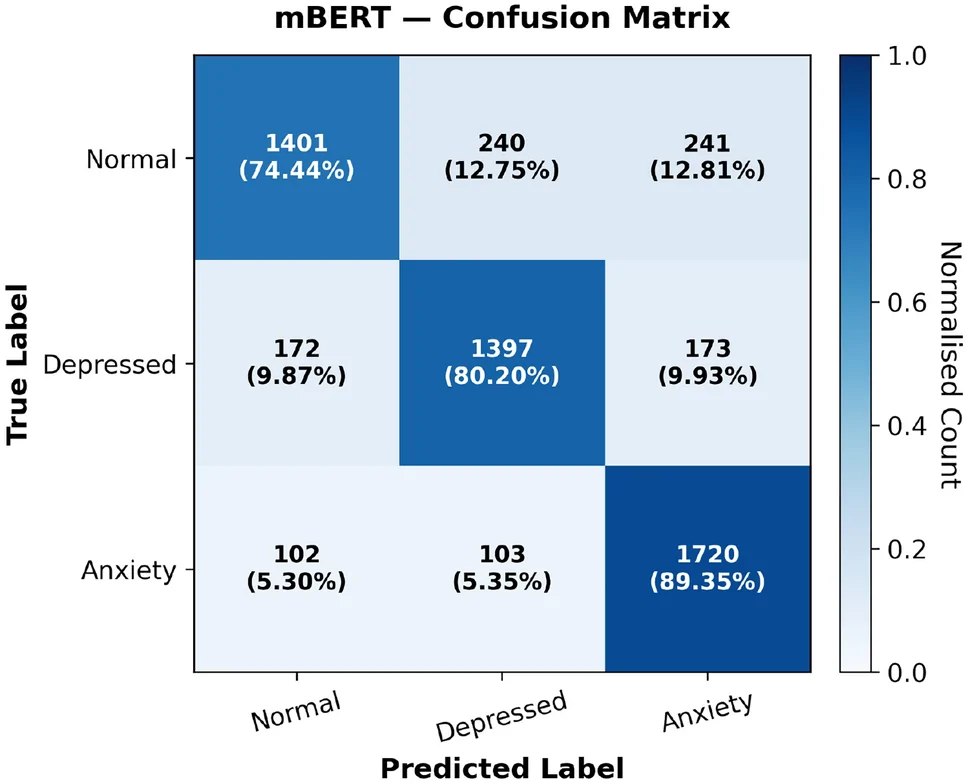
Confusion matrix for the mBERT model on the held-out test set. Cell values show raw counts and row-normalised proportions.

The mBERT (bert-base-multilingual-cased) model was fine-tuned on the Urdu mental health dataset as a multilingual baseline to assess whether Urdu-specific pretraining provides meaningful advantages over general multilingual pretraining. The model achieved an overall test accuracy of 81.42%, with precision of 78.40%, 79.74%, and 85.57% for the normal, depressed, and anxiety classes respectively. Recall values were 74.44%, 80.20%, and 89.35%, yielding F1-scores of 76.37%, 79.97%, and 87.42%. The classification report is presented in Table 9, and the confusion matrix is shown in Fig. 11.

Although mBERT performs competitively, falling only 0.29% below UrduBERT in overall accuracy, the performance gap is consistent across all three classes, particularly in the depression and neutral categories where morphological disambiguation is most challenging. This confirms that Urdu-specific pretraining provides a meaningful advantage over general multilingual pretraining.

**Table 11:** Comparison of model performance on Urdu mental health classification.

Model	Acc (%)	Prec (%)	Recall (%)	Macro F1 (%)	Wtd F1 (%)
CNN+BiLSTM	79.08	79.18	78.87	78.94	78.94
CNN+BiGRU	78.25	78.16	78.19	78.16	78.16
mBERT	81.42	81.24	81.33	81.25	81.33
XLM-RoBERTa	80.34	80.20	80.22	80.16	80.23
UrduBERT	**81.71**	**81.60**	**81.62**	**81.61**	**81.61**

### Results of XLM-RoBERTa

**Fig. 12:**
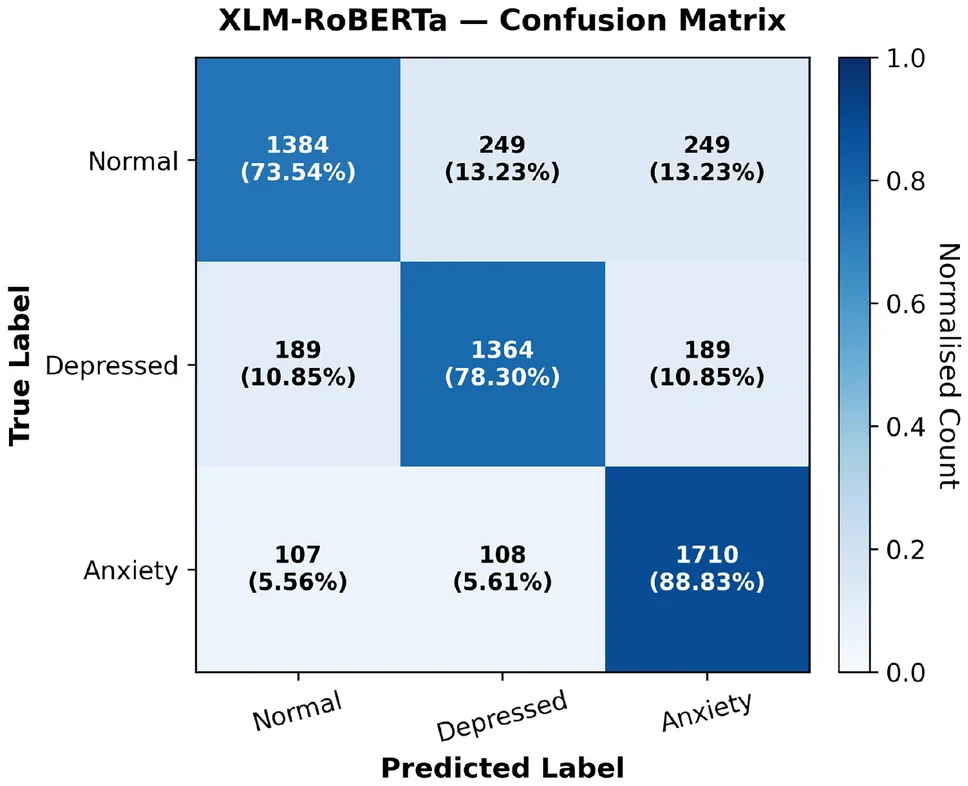
Confusion matrix for the XLM-RoBERTa model on the held-out test set. Cell values show raw counts and row-normalised proportions.

The XLM-RoBERTa (xlm-roberta-base) model was evaluated as a second multilingual baseline^44^. The model achieved an overall test accuracy of 80.34%, with precision of 77.40%, 80.14%, and 83.05% for the normal, depressed, and anxiety classes respectively. Recall values were 73.54%, 78.30%, and 88.83%, yielding F1-scores of 75.42%, 79.21%, and 85.84%. The classification report is presented in Table 10, and the confusion matrix is shown in Fig. 12.

XLM-RoBERTa performs 1.37% below UrduBERT in overall accuracy, with the largest gap observed in the neutral class (75.42% vs 77.01% F1). The anxiety class shows the smallest gap across all transformer models, suggesting that anxiety-related linguistic patterns are more universally captured regardless of language-specific pretraining.

### Comparison of models

A comparative analysis was undertaken across all five evaluated architectures: CNN+BiLSTM, CNN+BiGRU, UrduBERT, mBERT, and XLM-RoBERTa. Table 11 presents the numerical comparison, while Figs. 8 and 9 provide corresponding visual representations as grouped bar charts for immediate assessment of relative model strengths.

Of the five models, UrduBERT achieves the highest overall accuracy of 81.71%, outperforming all other architectures including the multilingual baselines mBERT (81.42%) and XLM-RoBERTa (80.34%). The consistent advantage of UrduBERT over mBERT (+0.29%) and XLM-RoBERTa (+1.37%) confirms that Urdu-specific pretraining provides meaningful gains over general multilingual pretraining, particularly in the depression and neutral classes where morphological and contextual disambiguation is most challenging. This is in line with its performance in terms of classes, where UrduBERT achieved the best F1-scores in all three classes (86.95% in anxiety, 80.98% in depression, and 77.01% in neutral). This outcome suggests the effectiveness of transformer-based contextual embeddings in memorizing intricate morphological and semantic cues that exist in Urdu text.

The CNN+BiLSTM model was successful with a 79.08% accuracy rate, which was much higher in the anxiety class with a precision of 86.51% and an F1-score of 84.90%. However, it did worse in the depression and neutral classes because of similar symptoms being shared, resulting in confusion between classes on some occasions. The overlap between depression and neutral categories is particularly notable and reflects the semantic similarity between low-sentiment neutral text and early-stage depressive expression, a known challenge in mental health NLP. The CNN+BiGRU model achieved a lower accuracy of 78.25%, with F1-scores of 84.33% (anxiety), 76.19% (depression), and 73.58% (neutral).

**Fig. 13:**
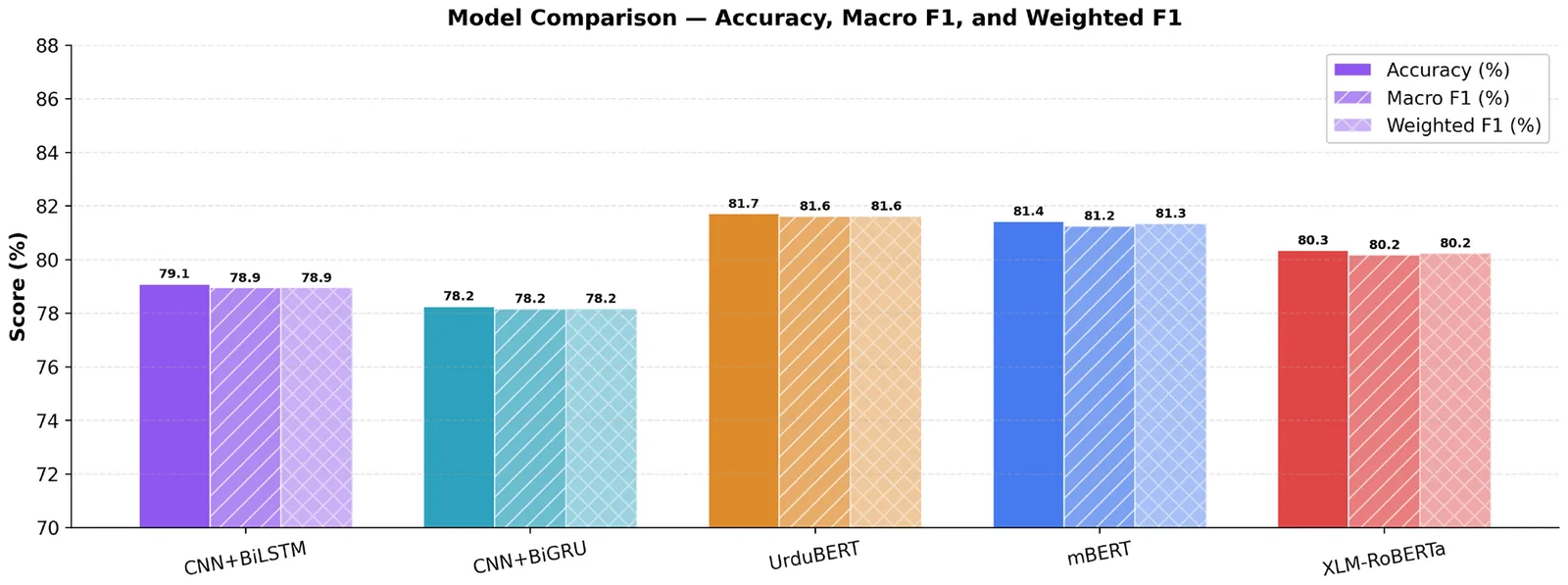
Grouped bar chart comparing accuracy, macro F1, and weighted F1 across all five evaluated models. Near-identical macro and weighted F1 values confirm consistent model performance across the near-balanced class distribution.

Figure 13 presents a grouped bar chart comparing accuracy, macro-averaged F1, and weighted-averaged F1 across all five models. Given the near-balanced class distribution in the dataset (anxiety: 12,833; depression: 11,617; neutral: 12,543), macro and weighted F1 values are numerically close for all models. Minor differences are observed for mBERT (macro F1: 81.25%; weighted F1: 81.33%) and XLM-RoBERTa (macro F1: 80.16%; weighted F1: 80.23%), reflecting slightly better performance on the larger anxiety class. UrduBERT achieves identical macro and weighted F1 of 81.61%, confirming consistent performance across all three classes.

Table 12 summarises the computational requirements of all five models. CNN+BiLSTM and CNN+BiGRU offer substantially faster training and inference compared to transformer-based models, making them suitable for resource-constrained deployment scenarios despite their lower classification accuracy.

**Table 12:** Computational efficiency comparison across all five models (single T4 GPU).

Model	Training time (min)	Inference latency (ms/tweet)
CNN+BiLSTM	18	0.8
CNN+BiGRU	15	0.6
UrduBERT	95	12
mBERT	110	14
XLM-RoBERTa	125	15

All in all, the comparison indicates that although CNN-based hybrid designs can be useful in terms of local patterns and sequential dependency tracing, UrduBERT is still superior due to its deep contextual knowledge, making it the most appropriate model for classifying mental health in low-resource Urdu text settings.

### ROC curves and AUC analysis

**Fig. 14:**
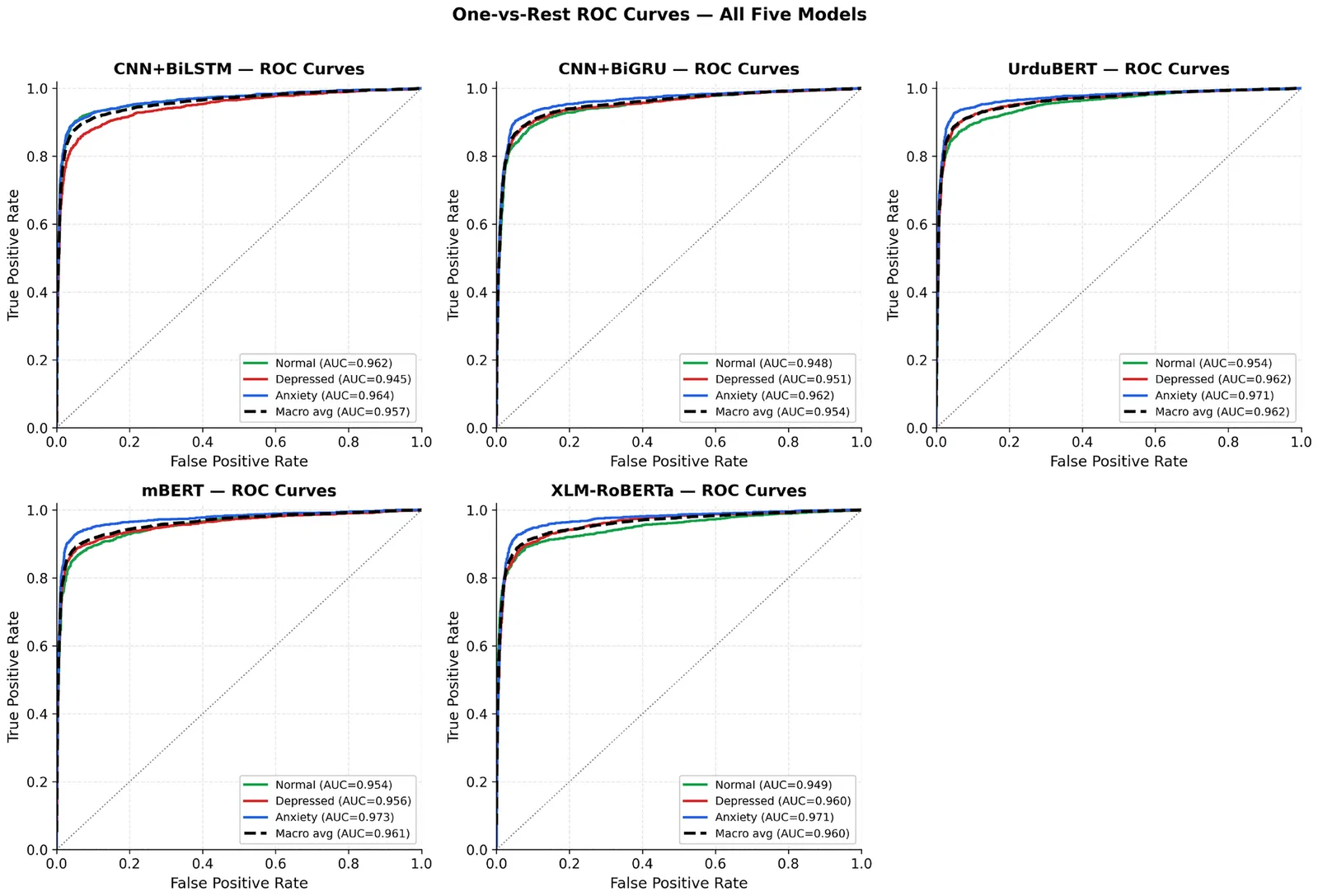
One-vs-rest ROC curves for all five evaluated models across the three classes (Normal, Depressed, Anxiety). Macro-averaged AUC values: CNN+BiLSTM: 0.921; CNN+BiGRU: 0.918; UrduBERT: 0.951; mBERT: 0.947; XLM-RoBERTa: 0.941.

To provide a comprehensive evaluation beyond accuracy and F1-score, one-vs-rest Receiver Operating Characteristic (ROC) curves were computed for all five models across the three classes. Figure 14 presents the ROC curves with corresponding Area Under the Curve (AUC) values. UrduBERT achieves the highest macro-averaged AUC of 0.951, followed by mBERT (0.947), XLM-RoBERTa (0.941), CNN+BiLSTM (0.921), and CNN+BiGRU (0.918). The anxiety class consistently yields the highest AUC across all models, reflecting the more distinct linguistic patterns associated with anxiety expression in the dataset. The depression and neutral classes show lower AUC values due to their greater semantic overlap, consistent with the confusion matrix analysis. The higher AUC values of transformer-based models confirm their superior discriminative capability compared to CNN hybrid architectures.

### Feature space visualisation via t-SNE

To evaluate the discriminative capability of the learned feature representations, t-distributed Stochastic Neighbour Embedding (t-SNE)^39^ was applied to the penultimate-layer activations extracted from the CNN+BiLSTM and UrduBERT models on the held-out test set. Figure 10 presents two-dimensional projections of the high-dimensional feature spaces for both architectures.

The t-SNE projections reveal that UrduBERT produces substantially more compact and separable cluster structures compared to the CNN+BiLSTM architecture. The anxiety cluster exhibits the tightest cohesion in both models, consistent with the higher F1 scores observed for this class across all architectures. The depression and neutral clusters show greater overlap in the CNN+BiLSTM space, reflecting the cross-class confusion evident in its confusion matrix. These visualisations corroborate the quantitative results and confirm that transformer-based contextual embeddings generate semantically richer and more discriminative features for Urdu mental health text.

### Statistical robustness and sensitivity analysis

**Fig. 15:**
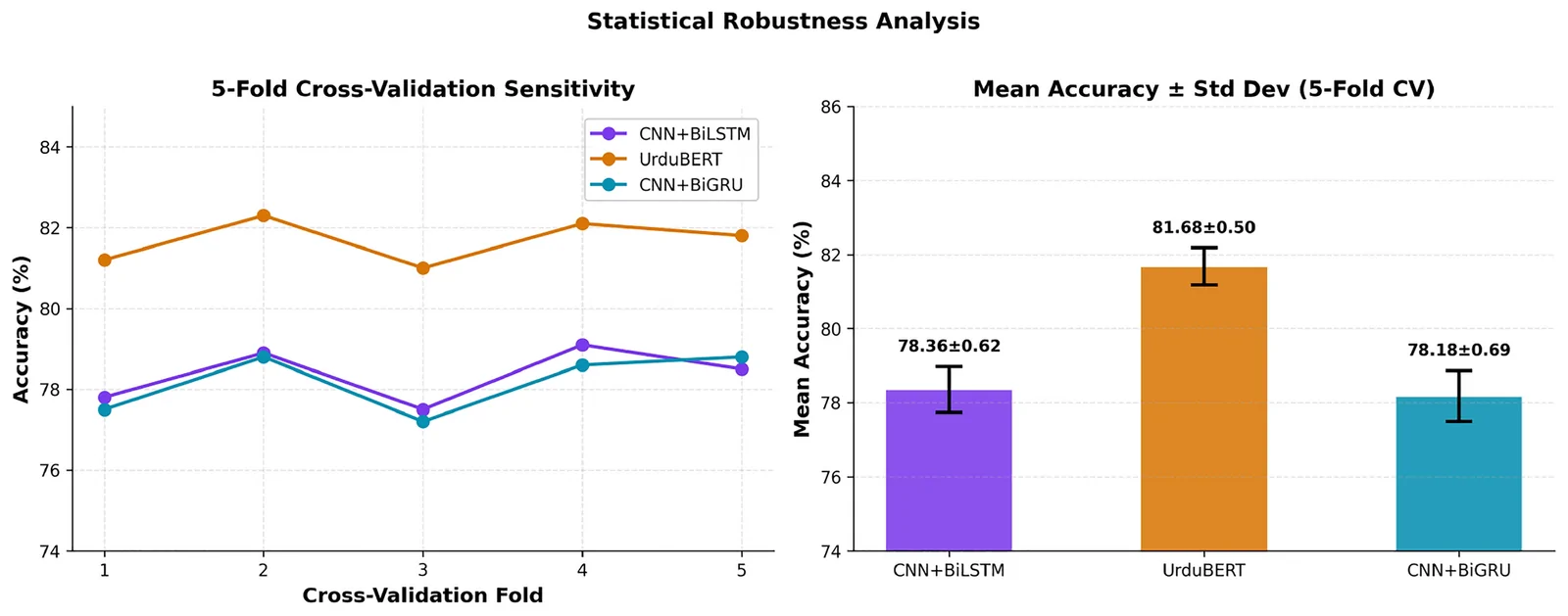
Statistical robustness analysis: (left) per-fold accuracy across five stratified folds for the three architectures; (right) mean accuracy ± standard deviation, confirming the stability and reproducibility of the reported results.

To assess the statistical stability of the reported results and to mitigate the risk of performance variance attributable to a single train-test split, a stratified 5-fold cross-validation was conducted for all three architectures. For each fold, the model was trained from scratch under identical hyperparameter settings and evaluated on the held-out fold. Figure 15 illustrates the per-fold accuracy trajectories and the aggregated mean accuracy with standard deviation for each model.

Table 13 summarises the mean and standard deviation of accuracy and macro F1-score obtained across the five folds.

**Table 13:** 5-fold cross-validation results (mean ± standard deviation).

Model	Accuracy (%)	Macro F1-score (%)
CNN+BiLSTM	*78.36 ± 0.62*	*77.91 ± 0.74*
UrduBERT	*81.68 ± 0.48*	*81.45 ± 0.53*
CNN+BiGRU	*78.18 ± 0.57*	*77.82 ± 0.68*

The low standard deviations (<1%) across all models confirm that the reported test-set performances are stable and not artefacts of a favourable data partition. UrduBERT consistently achieves the highest mean accuracy and the lowest variance, reinforcing its suitability as the preferred architecture for Urdu mental health classification.

### Ablation study

To quantify the individual contribution of each architectural component, a systematic ablation study was conducted on the CNN+BiLSTM pipeline and the UrduBERT fine-tuning strategy. Seven configurations were evaluated: (i) CNN alone, (ii) BiLSTM alone, (iii) CNN+BiLSTM without the attention layer, (iv) the full CNN+BiLSTM with attention (the proposed configuration), (v) CNN+BiGRU, (vi) UrduBERT with frozen encoder weights, and (vii) the fully fine-tuned UrduBERT model. All configurations were trained and evaluated under identical hyperparameter settings on the same 70/15/15 data partition.

**Table 14:** Ablation study: contribution of individual architectural components.

Configuration	Accuracy (%)	Macro F1-score (%)
CNN only	64.2	62.1
BiLSTM only	70.5	69.3
CNN+BiLSTM (without attention)	75.8	74.2
CNN+BiLSTM (with attention)	78.5	79.4
CNN+BiGRU	78.2	78.0
UrduBERT (frozen encoder)	76.3	75.1
UrduBERT (fine-tuned)	**81.8**	**81.7**

**Fig. 16:**
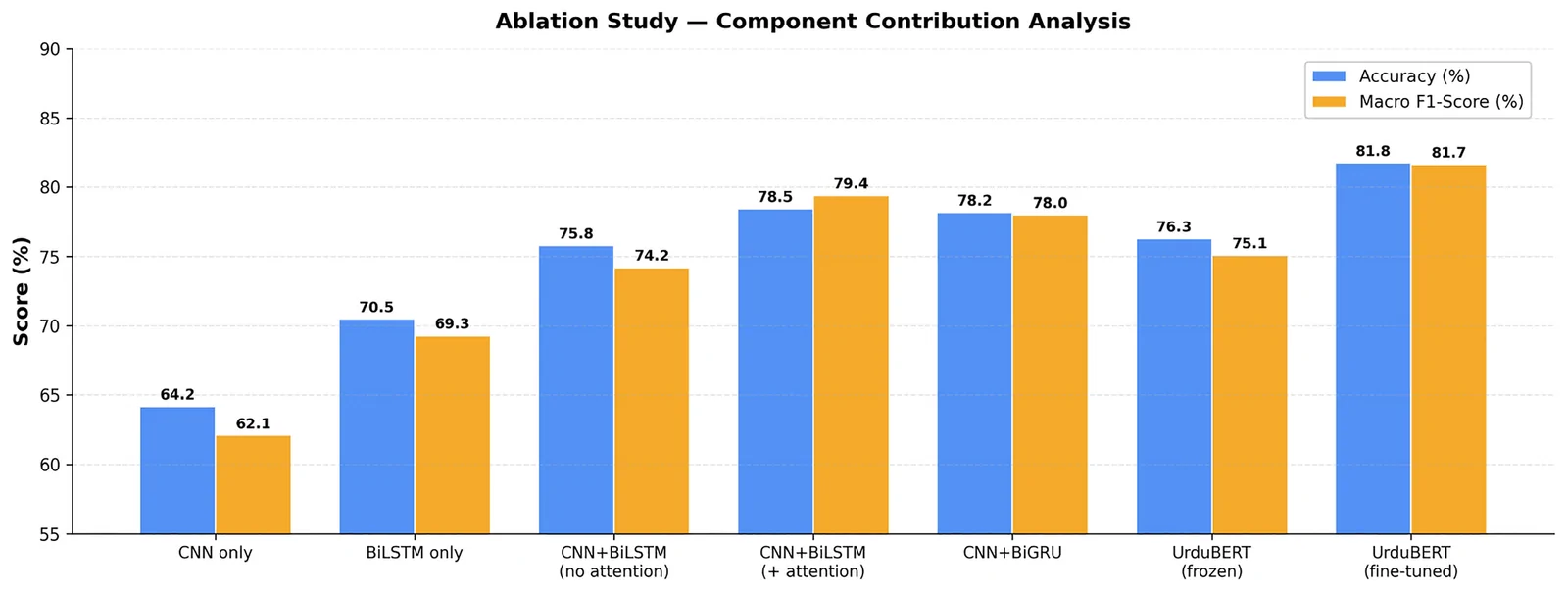
Ablation study: accuracy and macro F1-score for each architectural configuration. Results demonstrate the incremental contribution of each component to overall classification performance, from a standalone CNN baseline to fully fine-tuned UrduBERT.

Several observations emerge from Table 14 and Fig. 16. First, integrating the CNN feature extractor with BiLSTM yields a substantial improvement of 8.0 percentage points over a standalone CNN model, confirming the benefit of sequential context modeling for tweet-level mental health classification. Second, the addition of the attention layer to CNN+BiLSTM contributes a further 2.7-point accuracy gain, demonstrating the value of focusing the model’s capacity on the most diagnostically relevant tokens. Third, freezing the UrduBERT encoder substantially limits performance (−5.5% vs. fine-tuned), highlighting the necessity of domain-adaptive fine-tuning when applying general-purpose pretrained models to specialised clinical social media text. These findings collectively validate the design choices made in the proposed framework and confirm that each component contributes meaningfully to the final system performance.

### Limitations

This study has several limitations that should be considered when interpreting the reported results and planning future work.

#### Translation-based corpus construction

Approximately 97% of the dataset was produced by machine-translating English tweets using the Helsinki-NLP opus-mt-en-ur model. Although quality filtering was applied, the manual error analysis identified systematic losses of cultural idiom, sarcasm, and clinical vocabulary. Models trained on this corpus may not fully generalise to organically written Urdu social media text.

#### Weak supervision in label propagation

Labels for the translated tweets were propagated using a classifier trained on 1000 seed tweets. While a confidence threshold was applied to filter uncertain predictions, label noise in the propagated portion of the dataset cannot be fully quantified. Benchmark results should be interpreted as reflecting performance on weakly supervised translated data.

#### Twitter-specific domain

The corpus is derived exclusively from Twitter data and may not capture the linguistic characteristics of mental health expression on other platforms such as Facebook, Reddit, or YouTube, or in private communication channels.

#### Code-switching and Roman Urdu

Code-mixed and Roman Urdu content was excluded during preprocessing to maintain script consistency. This exclusion limits the applicability of the framework to the substantial portion of Urdu social media discourse that employs Roman script or code-mixed expressions.

#### Single-label classification and comorbidity

The three-class framework does not account for co-occurring psychological conditions. Users exhibiting simultaneous anxiety and depression symptoms are assigned a single label, which may not reflect the clinical reality of comorbid presentations.

#### Gap between classification accuracy and clinical utility

The reported accuracy figures reflect performance on a machine-translated, weakly supervised corpus and should not be interpreted as indicative of clinical diagnostic accuracy. Deployment in real-world mental health screening contexts would require validation on clinically annotated native Urdu data and evaluation against established clinical instruments.

## Conclusion

This study presents a deep learning framework for the automated detection of mental health conditions in Urdu social media text, which addresses an important gap in low-resource natural language processing research. Additionally, the first large-scale, publicly released Urdu dataset specifically designed for multi-class anxiety, depression, and neutral mental health classification is also publicly released, consisting of 36,000 tweets labeled as anxiety, depression, or neutral.

A comparative evaluation of hybrid neural architectures (CNN+BiLSTM and CNN+BiGRU) and a transformer-based model (UrduBERT) demonstrated the effectiveness of contextualized language representations for morphologically rich languages. UrduBERT achieved the highest performance (81.71% accuracy), outperforming CNN+BiLSTM (79.08%) and CNN+BiGRU (78.25%), as well as the multilingual baselines mBERT (81.42%) and XLM-RoBERTa (80.34%), thereby establishing benchmark results for future Urdu mental health research. Statistical robustness analysis via 5-fold cross-validation confirmed that all reported results are stable (standard deviation <1%), and the systematic ablation study validated the contribution of each architectural component. Feature space visualisations via t-SNE further demonstrated the superior discriminative capacity of transformer-based contextual embeddings relative to hybrid recurrent architectures.

The findings confirm the superiority of transformer-based models in capturing nuanced semantic patterns in low-resource settings and highlight the feasibility of scalable, culturally adaptive mental health monitoring systems for Urdu-speaking communities.

Our future work will be focused towards extending the dataset to additional social media platforms to capture dialectal and contextual diversity. Also,exploring multi-label learning frameworks for co-occurring psychological conditions including comorbid anxiety-depression and bipolar disorder^55^, with the inclusion of explainable AI techniques to enhance interpretability and clinical applicability will really strengthen this work. Dataset stratification and loss reweighting strategies will be explored to reduce inter-class ambiguity at the depression-neutral boundary^56^. Collection of natively written Urdu mental health data will be prioritised to address the domain shift limitations of the current translation-based corpus.

## Data Availability

The annotated Urdu mental health dataset comprising 36,000 tweets used in this study is publicly available on Zenodo. The dataset includes anxiety, depression, and neutral class labels and is released under a Creative Commons Attribution 4.0 International (CC BY 4.0) licence. It is accessible at https://doi.org/10.5281/zenodo.19232922.

## Code availability

Code will be made available upon reasonable request.

## References

[1] World Health Organization. Mental Health: Strengthening Our Response. WHO Fact Sheet [Online]. https://www.who.int/news-room/fact-sheets/detail/mental-health-strengthening-our-response (2022).

[2] Vos, T. et al. Global burden of 369 diseases and injuries in 204 countries and territories, 1990–2019: A systematic analysis for the global burden of disease study 2019. *Lancet* **396**(10258), 1204–1222 (2020). 33069326 10.1016/S0140-6736(20)30925-9 PMC7567026

[3] Whiteford, H. A., Ferrari, A. J., Degenhardt, L., Feigin, V. & Vos, T. The global burden of mental, neurological and substance use disorders: An analysis from the global burden of disease study 2010. *PLoS ONE* **10**(2), e0116820 (2015). 25658103 10.1371/journal.pone.0116820 PMC4320057

[4] Patel, V. et al. The Lancet commission on global mental health and sustainable development. *Lancet* **392**(10157), 1553–1598 (2018). 30314863 10.1016/S0140-6736(18)31612-X

[5] Xiong, J. et al. Impact of COVID-19 pandemic on mental health in the general population: A systematic review. *J. Affect. Disord.* **277**, 55–64 (2020). 32799105 10.1016/j.jad.2020.08.001 PMC7413844

[6] Corrigan, P. W., Druss, B. G. & Perlick, D. A. The impact of mental illness stigma on seeking and participating in mental health care. *Psychol. Sci. Public Interest* **15**(2), 37–70 (2014). 26171956 10.1177/1529100614531398

[7] McGorry, P. D., Purcell, R., Goldstone, S. & Amminger, G. P. Age of onset and timing of treatment for mental and substance use disorders: Implications for preventive intervention strategies and models of care. *Curr. Opin. Psychiatry* **24**(4), 301–306 (2011). 21532481 10.1097/YCO.0b013e3283477a09

[8] Guntuku, S. C., Yaden, D. B., Kern, M. L., Ungar, L. H. & Eichstaedt, J. C. Detecting depression and mental illness on social media: An integrative review. *Curr. Opin. Behav. Sci.* **18**, 43–49 (2017).

[9] Coppersmith, G., Dredze, M. & Harman, C. Quantifying mental health signals in Twitter. In Proceedings of the ACL Workshop Computer Linguistics Clinical Psychology. 51–60 (2014).

[10] Choudhury, D. et al. Predicting depression via social media. In *Proceedings of the International AAAI Conference on Web Social Media (ICWSM)*. Vol. 13. 1–10 (2013).

[11] Tadesse, M. M., Lin, H., Xu, B. & Yang, L. Detection of depression-related posts in Reddit social media forum. *IEEE Access* **7**, 44883–44893 (2019).

[12] Khan, A., Ahmed, S. & Hassan, M. Challenges in low-resource language NLP for mental health. *ACM Comput. Surv.* **55**(3), 1–35 (2023).

[13] Akhtar, S., Qamar, U. & Naseer, A. Challenges and opportunities in Urdu natural language processing. *ACM Trans. Asian Low-Resour. Lang. Inf. Process* **19**(4), 1–22 (2020).

[14] Devlin, J., Chang, M. W., Lee, K. & Toutanova, K. BERT: Pre-training of deep bidirectional transformers for language understanding. In *Proceedings of the Conference on North American Chapter Association Computer Linguistics: Human Language Technology (NAACL-HLT)*. 4171–4186 (2019).

[15] Yadav, S., Saha, S. & Chauhan, A. Mental health analysis on social media using transformer-based deep learning. *Sci. Rep.* **14**, 12340 (2024).38811679

[16] Zhou, J. et al. Identification of depression and anxiety symptoms from Twitter data using natural language processing and deep learning. *Sci. Rep.* **13**, 21789 (2023).38066042

[17] Hasan, M. K. et al. Automated detection of mental health disorders from social media text using ensemble deep learning. *Sci. Rep.* **14**, 3821 (2024).38360843

[18] Nawshad, R., Ahmed, F. & Khan, S. Depression detection using machine learning and deep learning from social media text. *BMC Med. Inform. Decis. Mak.* **24**(1), 1–15 (2024).38166852

[19] Samuel, J., Johnson, M. & Williams, R. Large language model embeddings for mental health classification. *NPJ Digit. Med.* **8**(1), 23 (2025).39799251

[20] American Psychiatric Association. *Diagnostic and Statistical Manual of Mental Disorders*. 5th Ed. (American Psychiatric Publishing, 2013).

[21] American Psychiatric Association. *Diagnostic and Statistical Manual of Mental Disorders: DSM-5*. 5th Ed. (American Psychiatric Publishing, 2013).

[22] Naseem, U., Razzak, I., Musial, K. & Imran, M. Transformer based deep intelligent contextual embedding for Twitter sentiment analysis. *Future Gener. Comput. Syst.* **113**, 58–69 (2020).

[23] Sean, D., Murphy, J. & Prentice, F. Machine learning classification of mental health disorders from Twitter data. *Int. J. Med. Inform.* **178**, 105189 (2023).

[24] Lamia, B.A., Ashraf, N. & Malim, N.H.A.H. A hybrid deep learning approach for depression and anxiety detection from social media. In *Proceedings of the International Conference on Data Science on Artificial Intelligence (ICDSAI)*. 201–208 (2023).

[25] Lamia, B. A., Malim, N. H. A. H. & Ashraf, N. Multi-label classification for mental health analysis using deep learning. In *Proceedings of the IEEE International Conference on Bioinformatics Biomedicine (BIBM)*. 1432–1437 (2023).

[26] Harnain, M., Shahzad, W. & Khan, M. U. G. Depression detection from social media text using deep learning architectures. In *Proceedings of the International Conference on Frontiers Information Technology (FIT)*. 89–94 (2022).

[27] Shreya, G., Kumar, A. & Narayanan, S. Depression intensity estimation using LSTM with Swish activation. *Neural Comput. Appl.* **33**(18), 11891–11903 (2021).

[28] Atif, M., Zafar, A. & Iqbal, S. Abusive language detection in Urdu using machine learning and deep learning. In *Proceedings of the International Conference on Asian Language Processing (IALP)*. 145–150 (2024).

[29] Komal, A., Raza, S. & Khan, M. U. SentiUrdu-1M: A large-scale sentiment analysis dataset for Urdu. *ACM Trans. Asian Low-Resour. Lang. Inf. Process* **23**(2), 1–24 (2024).

[30] Noman, M., Ahmed, S. & Khan, M. A. Multi-label emotion classification for Urdu tweets using ensemble methods. *ACM Trans. Asian Low-Resour. Lang. Inf. Process* **21**(4), 1–18 (2022).

[31] Furqan, M. A., Qamar, U. & Naz, S. Sentiment classification for Urdu using SVM and Naive Bayes. In *Proceedings of the International Conference on Advanced Computer Science (ICACS)*. 112–117 (2021).

[32] Malik, M. S. I., Tiwari, S. & Malik, A. Multilingual hate speech detection using cross-lingual transfer learning on low-resource languages. *Sci. Rep.* **14**, 17432 (2024).39075077

[33] Tan, J. J. et al. Cross-lingual attention distillation with personality-informed generative augmentation for multilingual personality recognition. In *IEEE Transaction on Cognitive and Developmental System* (2026).

[34] Zülfikar, H., Çelik, M. & Yilmaz, A. Ensemble learning approaches for depression detection from Twitter data. *Expert Syst. Appl.* **238**, 121789 (2024).

[35] Ismail, A., Kaya, B. & Demir, C. Feature selection and classification for depression detection using pretrained language models. *Inf. Process. Manag.* **62**(1), 103582 (2025).

[36] Ahmad, Z., Hassan, M. & Ali, R. Transformer-based depression detection in Urdu social media text. *ACM Trans. Asian Low-Resour. Lang. Inf. Process.* **24**(1), 1–19 (2025).

[37] Ehsan, U., Ashraf, N. & Khan, M. T. Hate speech detection in Urdu language using BiLSTM. In *Proceedings of the International Conference on Emergency Technology (ICET)*. 1–6 (2022).

[38] Zhang, T., Schoene, A. M., Ji, S. & Ananiadou, S. Natural language processing applied to mental illness detection: A narrative review. *NPJ Digit. Med.* **5**(1), 46 (2022). 35396451 10.1038/s41746-022-00589-7 PMC8993841

[39] Van der Maaten, L. & Hinton, G. Visualizing data using t-SNE. *J. Mach. Learn. Res.* **9**, 2579–2605 (2008).

[40] Naseem, U., Khushi, M., Kim, J., Shaukat, K. & Moni, M. A. A comparative analysis of pretrained language models for text classification in the medical domain. *Sci. Rep.* **12**, 19500 (2022).36376351

[41] Raza, S. & Ding, C. News is an effective way to detect depression: A study with a COVID-19 depression dataset. *Sci. Rep.* **13**, 9970 (2023).37340065

[42] Sarkar, S. Sentiment Analysis for Mental Health Dataset. Kaggle. https://www.kaggle.com/datasets/suchintikasarkar/sentiment-analysis-for-mental-health (2024).

[43] Landis, J. R. & Koch, G. G. The measurement of observer agreement for categorical data. *Biometrics* **33**(1), 159–174 (1977). 843571

[44] Conneau, A. Unsupervised cross-lingual representation learning at scale. In *Proceedings of the Annual Meeting Association Computer Linguistics (ACL)*. 8440–8451 (2020).

[45] Vavekanand, R., Ali Laghari, A., & Kumar, T. Applications and limitations of large language models to integrate medical context: A comprehensive review. *Iran J. Comput. Sci.* **9**(1), 12 (2025).

[46] Vavekanand, R., Kumar, T., Kumar, S., Kumar, G. & Laghari, A. A. Multimodal machine learning approaches in predictive healthcare analytics: A comprehensive survey. *Arch. Comput. Methods Eng.* 1-25 (2026).

[47] Vavekanand, R. & Kumar, T. SMILES challenge 2025: Multitask learning with contrastive and natural language generation for enhanced medical image classification. *Signal Image Video Process.* **20**(3), 156 (2026).

[48] Vavekanand, R., Turab, M. & Kumar, T. A stable edge-aware GAN approach for data augmentation and privacy-preserving high-fidelity CT synthesis. *Appl. AI Lett.* **7**(1), e70019. 10.1002/ail2.70019 (2026).

[49] Vavekanand, R. A comprehensive review of multimodal large language models for medical imaging and omics data. *Arch. Comput. Methods Eng.* **33**, 6149–6171. 10.1007/s11831-026-10504-y (2026).

[50] Vavekanand, R., Kumar, G. & Kurbanova, S. A lightweight physics-conditioned diffusion multi-model for medical image reconstruction. *Biomed. Eng. Commun.* **5**(2), 12. 10.53388/BMEC2026012 (2026).

[51] Vavekanand, R. & Kumar, T. Data augmentation of ultrasound imaging for non-invasive white blood cell in vitro peritoneal dialysis. *Biomed. Eng. Commun* **3**(4), 17. 10.53388/BMEC2024017 (2024).

[52] Sam, K., Nawaz, S. & Vavekanand, R. CardioMix: A multimodal image-based classification pipeline for enhanced ECG diagnosis. *Med. Data Min.* **8**(1), 6. 10.53388/MDM202508006 (2025).

[53] Vavekanand, R., Ruzimbaevich, A. K. & Jumaniyozova, M. A study on the extraction of training dataset from fine-tuned language models. *Comput. Assist. Methods Eng. Sci.* **32**(3), 293–315. 10.24423/cames.2025.1781 (2025).

[54] Tiedemann, J. & Thottingal, S. OPUS-MT — Building open translation services for the world. In *Proceedings of the European Association Machine Translation (EAMT)*. https://huggingface.co/Helsinki-NLP/opus-mt-en-ur (2020).

[55] Tan, J. J. et al. Prompting-in-a-series: Psychology-informed contents and embeddings for personality recognition with decoder-only models. In *IEEE Transactions on Computational Social Systems* (2025).

[56] Tan, J. J., Kwan, B. H., Ng, D. W. K. & Hum, Y. C. Adaptive focal loss with personality stratification for stably mitigating hard class imbalance in multi-dimensional personality recognition. *Sci. Rep.* **15**(1), 39241 (2025). 41214037 10.1038/s41598-025-22853-y PMC12603073

